# P19 Disparities in infection, antimicrobial resistance and consumption rates: findings from national reporting in England 2018–23

**DOI:** 10.1093/jacamr/dlae217.023

**Published:** 2025-01-23

**Authors:** Ellie L Gilham, Hannah Higgins, Emily Mason, Jacquelin McCormick, Katherine L Henderson, Mariyam Mirfenderesky, Rebecca Oettle, Berit Muller-Pebody, Alicia Demirjian, Russell Hope, Colin Brown, Diane Ashiru-Oredope

**Affiliations:** UK Health Security Agency, London, UK; UK Health Security Agency, London, UK; UK Health Security Agency, London, UK; UK Health Security Agency, London, UK; UK Health Security Agency, London, UK; UK Health Security Agency, London, UK; UK Health Security Agency, London, UK; UK Health Security Agency, London, UK; UK Health Security Agency, London, UK; UK Health Security Agency, London, UK; UK Health Security Agency, London, UK; UK Health Security Agency, London, UK; School of Pharmacy, University of Nottingham, Nottingham, UK

## Abstract

**Background:**

In 2019, the UKHSA established an antimicrobial resistance (AMR) health inequalities (HI) workstream, with one aim of the workstream being to improve AMR and antimicrobial consumption (AMC) surveillance to understand burden and trends of AMR and AMC for Core20PLUS populations. UKHSA also published its Health Equity Strategy in 2023.

**Objectives:**

To highlight changes in national reporting of bloodstream infections (BSI), resistant BSIs and AMC by HI factors and to summarize data on HI included within the 2023–24 English Surveillance Programme for Antimicrobial Utilisation and Resistance (ESPAUR) report.

**Methods:**

Changes in HI data reporting were reviewed over the 10 years of ESPAUR. BSI and AMR data were obtained from UKHSA’s Second-Generation Surveillance System; primary and secondary care prescribing data were obtained from the UKHSA Antibiotic Prescribing Data Warehouse sourced from NHS Business Services Authority and IQVIA, respectively between 2018 and 2023. Poisson regression models were used to examine the relationship between AMR burden rates and IMD quintile levels and years.

**Results:**

Prior to 2021 reporting of factors associate with HI was limited with rates of BSIs, AMR and AMC only reported by age and region. Since 2021–22, data on resistant BSIs by ethnic group and index of multiple deprivation (IMD) has been included. Furthermore, in 2023–24 data on AMC by IMD has also been included. The 2023–24 ESPAUR report showed regional variation, as well as differences by age, ethnicity and IMD for BSIs and AMR, and by age, IMD and region for AMC. The highest percentage resistance of BSIs was seen in Asian and British Asian ethnic groups (39.4%; 95% CI: 33.2–36.1), almost double that seen in white populations (20.1%). Rates of resistant BSIs were 42.6% higher in the lowest compared with the highest IMD quintile in 2023 (38.1 versus 26.7 infections/100 000 population); (*P<*0.005), an increase of 13.2% from 2019. While rates of BSI were highest in the Northeast (186.5/100 000 population), the burden of resistant infections was highest in London (41.5/100 000 population) compared with the Southwest which had the lowest burden of resistant infections (25.9/100 000). Antibiotic prescription rates varied by age group with prescribing highest amongst those aged 65-years and over (1.07 items prescribed per 1000 inhabitants/day). Between 2018 and 2023 prescribing has consistently been highest in the Northeast and lowest in London. London has much lower levels of primary care prescribing (10.9 DID [defined daily doses per 1000 inhabitants per day[), but the highest levels of secondary care prescribing (4.7 DID) compared with other regions. Finally, primary care prescribing was highest in the lowest compared with the highest IMD groups (25.0 versus 14.8 items per 1000 individuals per day).

**Conclusions:**

The 2023–24 ESPAUR report highlights high levels of variation in BSI rate, AMR and AMC, by geography, age, ethnicity and IMD, however the root causes of these inequalities can be difficult to determine. The dedicated HI outcome within the UK 2024–29 National Action Plan for AMR will support the development and evaluation of stewardship and engagement interventions to prevent infections and optimize AMC among populations with factors associated with HI.

